# Lean management in health care: definition, concepts, methodology and effects reported (systematic review protocol)

**DOI:** 10.1186/2046-4053-3-103

**Published:** 2014-09-19

**Authors:** Adegboyega K Lawal, Thomas Rotter, Leigh Kinsman, Nazmi Sari, Liz Harrison, Cathy Jeffery, Mareike Kutz, Mohammad F Khan, Rachel Flynn

**Affiliations:** 1 College of Pharmacy and Nutrition, University of Saskatchewan, Saskatoon S7N 5A2, Canada; 2 School of Rural Health, Monash University, Bendigo, Australia; 3 Department of Economics, University of Saskatchewan, Saskatoon, SK, Canada; 4 School of Physical Therapy, College of Medicine, University of Saskatchewan, Saskatoon, SK, Canada; 5 College of Nursing, University of Saskatchewan, Saskatoon, SK, Canada; 6 Faculty of economy, University of Applied Sciences, Osnabrueck, Germany; 7 School of Public Health, University of Saskatchewan, Saskatoon, SK, Canada; 8 Faculty of Nursing, University of Alberta, Edmonton, AB, Canada

**Keywords:** Lean, Systematic review, Health care, Toyota management system

## Abstract

**Background:**

Lean is a set of operating philosophies and methods that help create a maximum value for patients by reducing waste and waits. It emphasizes the consideration of the customer’s needs, employee involvement and continuous improvement. Research on the application and implementation of lean principles in health care has been limited.

**Methods:**

This is a protocol for a systematic review, following the Cochrane Effective Practice and Organisation of Care (EPOC) methodology. The review aims to document, catalogue and synthesize the existing literature on the effects of lean implementation in health care settings especially the potential effects on professional practice and health care outcomes. We have developed a Medline keyword search strategy, and this focused strategy will be translated into other databases. All search strategies will be provided in the review. The method proposed by the Cochrane EPOC group regarding randomized study designs, non-randomised controlled trials controlled before and after studies and interrupted time series will be followed. In addition, we will also include cohort, case–control studies, and relevant non-comparative publications such as case reports. We will categorize and analyse the review findings according to the study design employed, the study quality (low- versus high-quality studies) and the reported types of implementation in the primary studies. We will present the results of studies in a tabular form.

**Discussion:**

Overall, the systematic review aims to identify, assess and synthesize the evidence to underpin the implementation of lean activities in health care settings as defined in this protocol. As a result, the review will provide an evidence base for the effectiveness of lean and implementation methodologies reported in health care.

**Systematic review registration:**

PROSPERO CRD42014008853

## Background

Lean is a set of operating philosophies and methods that help create maximum value for patients by reducing waste and waits [1]. It aims to fundamentally change organization thinking and value, which ultimately leads to the transformation of organization behaviour and culture over time [2]. Based on the Toyota model, it focuses on how efficiently resources are being used and ask, 'what value is being added for the customer’ in every process [3]. Recently, the health care industry has demonstrated success in applying these principles in the United States, United Kingdom, Australia and now Canada [4]. Despite indications that lean is prevalent in health care, many authors regard its implementation to be pragmatic, patchy and fragmented [5]. The application of lean management in health care can also be holistic such as the transformation of an overall business strategy [2,6]. Although lean thinking originated from car making, research on its application and sustainability in health care is still limited [7]. Primary studies often lack appropriate concepts explicitly stated, research designs, appropriate analysis and outcome measures [7]. The majority of studies also reported on successful lean interventions, whereas little has been documented about the failed attempts or barriers to its implementation in health care [7]. It is therefore imperative to catalogue and synthesize the existing literature via a systematic review on the effects of lean implementation especially the potential effects on professional practice and health care outcomes in various settings.

### Review questions and objectives

The primary review question is as follows:

What are the effects of lean management in health care on professional practice and health care outcomes?

The secondary review questions are as follows:

(i.) What can we learn from the existing evidence on lean to better understand the various methodologies used and the experience in evaluating the impact?

(ii.) What are the differences in lean implementation, and can we explain how those differences might lead to different outcomes?

### Criteria for considering existing publications for this review

The systematic review will include all relevant studies according to the review questions and objectives. We will apply the electronic search strategy to identify all primary studies reporting on the effectiveness of lean and the different strategies used for implementation. We will extract and collate all of the concepts used to describe lean, how it is applied and the activities involved in the implementation process. We will not include editorial reports, animal studies, lean applications in other industries, teaching and investigations using self-reported outcomes.

### Types of publications/studies

The method proposed by the Cochrane Effective Practice and Organisation of Care (EPOC) group regarding randomized study designs (RCTs), non-randomised controlled trials (NRCTs), controlled before and after studies (CBA) and interrupted time series (ITS) will be included [8]. In addition, we will also consider cohort or panel (longitudinal) studies, case–control studies and relevant non-comparative publications such as case reports. A case report is a document that provides details about how a study was conducted and its subsequent findings. A panel study is a longitudinal study in which variables are measured on the same units over time.

### Types of institutions and participants

•All sectors of the health care system, including hospital care, primary care and rehabilitation

•All employees such as CEOs, health professionals, administrative staff and support staff

•Patients and their families

•Management, lean experts and key stakeholders

### Types of lean interventions reported

#### Definition

Lean is a set of operating philosophies and methods that help create a maximum value for patients by reducing waste and waits [1]. The approach was originally derived from the Toyota car company production line system: a continuous process improvement system comprising of structured inventory management, waste reduction and quality improvement techniques [9]. Lean utilises a continuous learning cycle that is driven by the 'true’ experts in the processes of health care, being the patients/families, health care providers and support staff [10].

The majority of lean investigations published in the international literature refer to the Toyota management system as applied to health care [11-18]. In particular, the Virginia Mason Medical Center’s application of lean 'became the catalyst for lean health care’ in other health systems, particularly in the United States and the United Kingdom [19,20]. Other authors refer to Thedacare [21] or simply to a lean management system or lean principles/lean philosophy [2,22-24].

### Lean application in Saskatchewan

In Saskatchewan, the Toyota lean management system is used in combination with a strategic management and policy deployment system, called Hoshin Kanri [25], and daily visual management. Daily visual management is an approach where staff members take the time each day to evaluate their progress using the key elements of daily huddles and visibility walls.

### Types of implementation strategy reported

Varying terms and Japanese terminologies are used to describe the lean implementation strategies. The most frequently reported lean implementation activities are 'lean basics’ workshops, also described as 'Kaizen basics’ workshops. A 'Kaizen or lean basics’ session is a one-day workshop, introducing lean tools and techniques [6,18]. Other activities reported in the literature to implement lean in health care are 5S events to reorganize the workplace, rapid process improvement workshops (RPIW) and value stream mapping to improve current and future care processes [11-13,26]. 5S stands for 'Sort, Sweep, Simplify, Standardize, Sustain/Self-Discipline’, and it represents a set of concepts that helps organizations ensure a clean and organized work place [27]. An RPIW is a week-long event also reported as a three-day lean event where teams of patients and their families, staff and clinicians focus on one problem, identify the root cause, create solutions and implement the solution in the workplace [27]. A value stream map in health care is a visual tool to understand the flow of patients, supplies or information through the journey of a patient, and it maps all processes required to deliver a health care service [27]. We will report on all activities used to implement lean concepts and methodologies. More examples of activities to be included are Kanban, lean leadership training, mistake-proofing projects, and other activities used to implement the lean management system. Kanban is a visual signalling system when new parts, supplies or services are needed, in the quantity needed, and at the time they are needed. A Kanban signal is usually a card, indicating the need to reorder supplies [27]. The aim of a mistake-proofing project is to develop a device or procedure to avoid such an error in the future (e.g. specific hose coupling in anaesthesia, forcing functions in order entry) [27].

### Types of outcome measures

All objectively reported process and outcome measures will be included. To be considered for this review, the studies must include one or more of the following primary or secondary outcomes.

#### Primary outcomes

Any objective measure of the following:

1. Health system improvement outcomes: admission time, collection time, turnaround time, triage time, time to see a physician, dispensing time, examination room time, number of patient visit, length of stay, discharge rate, patient journey time, scheduling time, near miss event rate, turnover time, wait time, etc.

2. Patient outcomes: patient satisfaction, mortality rate, re-admission rate, etc.

3. Professional outcomes: employee satisfaction, time spent with the patient, staff overtime, login to provider time, etc.

#### Secondary outcomes

Any objective measure of the following:

1. The various types of lean definitions or concepts: lean, lean philosophy, lean principles, continuous quality improvement, etc.

2. Lean management systems: Toyota management system, Henry ford production system, Thedacare improvement system, Virginia Mason production system, etc.

3. Lean activities: 5S, Value stream mapping, Rapid process improvement workshops, Kaizens basics workshops, 3P, etc.

### Search strategy for the identification of studies

To develop our search strategy, we ran the Medline search strategy (see Additional file 1, Strategy A) from a Cochrane review on the broad concept of continuous quality improvement [28]. However, this strategy was not focused on lean. Since lean is not represented in controlled vocabularies of biomedical databases, an information scientist developed a Medline search strategy (see Additional file 1, Strategy B) This focused keyword strategy will be translated and run in the databases listed below. We will not make use of methodological filters and will not apply date or language limits. All search strategies will be provided in the final review. The following electronic databases will be searched for primary studies: Medline (OVID), Embase (OVID), HealthStar (OVID), Web of Science (Science, Social Sciences, and Arts & Humanites Citations Indexes and Conference Proceedings), Health Technology Assessment (HTA), Economics Evaluation (EED) databases, Cochrane Library, EconLit, PAIS (Public Affairs Information Service) International, Proquest Dissertations & Theses, Proquest Political Science and Canadian Research Index (see Additional file 1, Strategy C).

#### Other search methods

We will also do the following:

•Search websites of organizations (grey literature searching) concerned with quality in health care such as AHRQ (Agency for Healthcare Research & Quality) and ASQ.org. Sites searched will be reported in the review.

•Contact the authors of relevant studies or reviews to clarify reported published information or to seek unpublished results/data (as needed).

•Contact researchers with expertise relevant to our topic (as needed).

•Conduct cited reference searches (in citation indexes) for studies we include in this review.

## Methods/Design

### Screening

All titles and abstracts will be included in a reference management database; duplicates will be deleted. Two review authors will independently screen all titles and abstracts (MFHK and MK) to assess which studies meet the inclusion criteria. We will retrieve the full text copies of all potentially relevant papers, and disagreement on the inclusion will be resolved by a third member of the research team (TR).

### Data management

We will record and report details on the number of retrieved references, the number of full text papers obtained and the number of included and excluded articles. We will manage this data in EndNote and use an excel spreadsheet. We will categorize articles based on three types of studies as suggested by a previously published literature review on lean management in hospitals [29]. The three article types are as follows: (1) articles that discuss the application of lean principles and are based only on the experience or general knowledge of the authors, (2) empirical articles based on actual case studies or research related to the application of lean principles and (3) literature reviews related to lean processes [29]. The reason for excluding retrieved full text studies will be stated in the final review.

### Data extraction

Pairs of two review authors (TR and LA, RF and MK, LH and NS, LK and CJ) will independently extract data according to the double data entry method by using a standardized data extraction sheet (Excel spreadsheet); they will extract data directly from the included studies. We will refer unresolved disagreements on data abstraction to a third review author (TR and LK) and if consensus cannot be reached, the contact author of the review, LA. If necessary, we will seek additional information from the authors of the primary studies.

### Risk of bias assessment

Two independent review authors will assess the methodological quality of all included studies, using the EPOC checklist for the assessment of methodological quality of studies [8]. EPOC criteria to be assessed include allocation of concealment, sequence generation, blinding of participants and personnel, similarities of baseline measures, confounding, similarities of baseline characteristics, management of incomplete outcome data, selective outcome reporting, contamination and other risk of bias identified by the review team. (See Additional file 2 for full list). For non-randomized designs such as case studies and cohort studies, we will use a tool for before-after studies that was developed based on the Newcastle-Ottawa scale [30] and used in a previous review [31]. Confounding factors (e.g. simultaneously ongoing initiatives such as changes in hospital policy and implementation of DRGs) will be also considered for case studies and cohort studies. The methodological quality of included studies will be assessed, and we will categorize them into three classes: A (low risk of bias), B (moderate risk of bias) and C (high risk of bias). We will refer unresolved disagreement on risk of bias to a third review author. We will consider studies with low risk of bias for all key domains or where it seems unlikely for bias to seriously alter the results. We will consider studies where risk of bias in at least one domain is unclear or judged to have some bias that could raise doubts about the conclusions as having an unclear risk of bias. We will consider studies with a high risk of bias in at least one domain or judged to have serious bias that decreases the certainty of the conclusions as having a high risk of bias [32]. We will not exclude studies from the review classified at high risk of bias. We will retain these studies and include them in a subsequent sensitivity analysis based on the assigned risk of bias.

### Data analysis and synthesis

For the primary review question, that is, the effects of lean management on professional practice and health care outcomes, data will be reported in natural units. For dichotomous data (i.e. odds ratio (OR) or risk ratio (RR)) we will calculate a crude event rate as a measure of overall frequency giving the total number of events occurring over the follow-up period reported unadjusted for covariates (i.e. sex, age). In the case of missing standard deviation, the appropriate transformation will be undertaken [32]. We will assess the data on resource use, costs and cost-effectiveness according to the methodology used in the individual studies [33]. Financial data will be presented in US$ for the same base year and will be adjusted for inflation by using a country-specific price index [34]. Additionally, we will provide the nominal cost data to allow readers to recalculate the results using alternative price indexes. Studies reporting in other currencies will be converted to US$ [35]. For the two secondary review questions, all relevant data will be extracted and presented in a tabular form. All outcomes will be counted and grouped in a tabular form into similar implementation activities, complications such as in-hospital complications and the direction of effect reported e.g. positive, negative and null. Relevant findings will be categorized and synthesized in the form of a narrative summary using text and evidence tables according to the definitions and implementation strategy reported in the primary study [36]. Whenever possible, we will attempt to contact the original investigators to request for missing information. For missing standard deviation, we will recalculate them from the reported statistics provided in these studies (e.g. confidence intervals, standard errors, *t* values, *P* values) [37].

### Combining studies

We will make an assessment of the reported lean methodologies, implementation strategies and effects, based upon the quality, size and direction of effects observed or reported. Positive, negative and null effects will be assessed, and studies will be grouped following the methods reported in the primary study. We will categorize and analyse the review findings according to the study design employed, the study quality (low- versus high-quality studies) and the method reported in the primary studies. The results will be presented in a tabular form. We expect to find both statistical and contextual heterogeneity, given the range of outcomes measured and the many different settings and types of professionals and patients included. This may make statistical pooling impossible, but if there seems be a group of studies amenable to meta-analysis, then a random-effect model will be employed with the results displayed graphically. We will assess statistical heterogeneity by visually inspecting the confidence intervals of the effect estimates and by calculating a test of heterogeneity (I squared test *I*^2^) [38], with a cut off of 60%.

### Subgroup analysis

We will perform a sub-group analysis of the primary and secondary outcomes reported where applicable. We will group studies according to the following categories:

1. Country(s) where the study was carried out (adjusting for possible market forces).

2. Setting(s) where the implementation of lean intervention occurred.

3. Year of publication to assess temporal differences in the outcomes reported over time

### Sensitivity analysis

Sensitivity analysis will be carried out to explore the robustness of the results by investigating the effects of including and excluding studies with high risk of bias and studies with missing information.

### Ongoing studies

We will describe identified ongoing studies, where available, detailing the primary author, research question(s), methods and outcome measures together with an estimate of the reporting date.

## Discussion

Overall, the systematic review aims to identify, assess and synthesize the evidence to underpin the various types of definition, concepts, methodology and effects of lean in health care settings as defined in this protocol. As a result, the review will provide an evidence base for the effectiveness of lean and the types of implementation strategies utilized, based on the review findings and conclusions.

## Competing interests

The authors declare that they have no competing interests.

## Authors’ contributions

All review authors have contributed to the production of the protocol, and all authors read and approved the manuscript. LA and TR led the writing of the protocol; all other review authors provided comment and feedback. For the full review, The Cochrane EPOC trail search coordinator, Michelle Fiander, has developed and will run the search strategy together with Vicky Duncan, the Nursing Liaison Librarian at the University of Saskatchewan. MK and MFHK will screen all the titles and abstracts for eligibility. TR and LA, RF and MK, LH and NS, LK and CJ will assess all primary studies for eligibility in review phase II. All review authors will abstract data, undertake analysis and write up the review. Michelle Fiander and VD will take the leadership regarding additional search strategies as defined in this review protocol. TR and NS will give advice on the methodological issues and the statistical analysis. LK would act as arbitrator should disagreement arise and will give advice on methodological issues. TR and LK will assess all full text studies in the second review stage about the practical relevance of the published methods. TR will lead the writing of the full review. LK and TR will critically appraise the review findings and conclusions, that is, to access the transferability of the international evidence.

## Supplementary Material

Additional file 1 **Search Strategies Lean Review.** This file contain details of the search strategy ran in a particular database (Medline). Click here for file

Additional file 2 **EPOC risk of bias criteria.** This file contains the suggested risk of bias for EPOC reviews. Click here for file
