# Supplementary material for: Lean management in health care: definition, concepts, methodology and effects reported (systematic review protocol)
Source: Syst Rev. 2014 Sep 19;3:103. doi: 10.1186/2046-4053-3-103 (PMC4171573; doi:10.1186/2046-4053-3-103)
Supplement: Additional file 1 — Search Strategies Lean Review. This file contain details of the search strategy ran in a particular database (Medline). [file 2046-4053-3-103-S1.pdf]

Medline search strategy for Lean management in healthcare: Definitions, concepts, Methodology and Effects reported (A systematic review protocol).

Search Strategies LEAN review (non-Cochrane)

A number of searches were conducted for this project.

Strategy A

In April 2013, the author team screened results of a sensitive search strategy written for a Cochrane systematic review: Brennan S, McKenzie JE, Whitty P, Buchan H, Green S, Bosch M. Continuous quality improvement: effects on professional practice and healthcare outcomes in primary care. *Cochrane Database of Systematic Reviews* 2009 , Issue 4 . Art. No.: CD003319. DOI: 10.1002/14651858.CD003319.pub2. Searches were run in Medline, EMBASE, CINAHL, and The Cochrane Library.

Strategy B: May 27, 2013

Conducted a focussed Medline keyword search in title and abstract which retrieved 338 citations. The authors selected 194 of these citations as relevant; and 8 as potentially relevant.

Strategy C: July 2013

Conducted a title keyword search based on analysis of 194 relevant citations identified by May 2013 search. Databases: Medline, Embase, HealthStar, Web of Science (Science, Social Sciences, and Arts & Humanites Citations Indexes; and Conference Proceedings), HTA and EED sections of the Cochrane Library, EconLit, PAIS International, Proquest Dissertations & Theses, Proquest Political Science, and Canadian Research Index.

Strategy A:
Medline search strategy for CQI Cochrane Review. The strategy was translated for other databases.

1. continuous quality improvement.ti,ab.
2. process improvement.ti,ab.
3. or/1-2 [CQI Set 1]
4. *Total quality management/ [This MeSH included CQI]
5. (quality adj3 (COLLABORATIVE? or circle? or team? or group?)).ti.
6. (TQM or CQI).ti,ab.
7. (quality adj2 improv$).ti. or (quality adj improv$).ab.
8. ("model for improv$" or "model to improve").ti,ab.
9. (collaborative adj2 improvement?).ti,ab.
10. (quality adj2 (COLLABORATIVE? or circle? or team? or group?)).ti,ab.
11. (breakthrough adj2 (series or collaborative or project?)).ti,ab.
12. total quality.ti,ab.
13. continuous quality.ti,ab.
14. continuous improvement.ti,ab.
15. quality management.ti,ab.
16. (PDSA or PDCA or TQIS or "plan do study" or "plan do check").ti,ab.
17. ((shewhart or shewart or deming) adj3 (cycle or method$)).ti,ab.
18. (rapid cycle or six sigma).ti,ab.
19. (breakthrough adj3 (series or project or collaborative?)).ti,ab.
20. (lean adj (approach or care or enterpri?e or management or method? or methodology or model? or oncology or philosophy or practice or practices or principles or program$ or thinking)).ab. or (lean and (approach or care or enterpri?e or management or method? or methodology or model? or oncology or philosophy or practice or practices or principles or program$ or thinking)).ti.
21. toyota.ti,ab. not automobiles/
22. ("concept map$" or "process map$" or "value stream map$" or "fault tree analysis" or "failure mode and effects analysis").ti,ab.
23. (Primary Health Care/ or General Practice/ or Family Practice/) and Quality Assurance, Health Care/ and (Quality Assurance, Health Care/ or quality.ti.)
24. or/4-23 [CQI Set 2]
25. Total quality management/
26. (improve? or improvement? or improving).ti.
27. (initiative? or outcome or outcomes or patient? or practice or primary care or service or services or system or systems).ti.
28. (implement$ or intervention$ or model?).ti.
29. ((primary adj2 care) or (community or outcome or outcomes or collaborat$ or team or teams)).ti,hw. or (practice or practices).ti. or practice.hw.
30. (structure? or structural or organi?ational$).ti.
31. care project?.ti.
32. (delivery and (service or services)).ti.
33. ((primary care or primary health care or primary healthcare) and improv$ and (care or management)).ti.
34. (primary care and (improve or improving) and (practice or practices)).ti.
35. (service? and implement$).ti.
36. (quality assurance adj4 (practice or practices or scheme or project? or implement$ or intervention$ or care or outcome or practitioner? or physician? or program? or programme or programmes or service or services)).ab.
37. (quality assurance and (practice or practices or scheme or project? or implement$ or intervention$ or care or outcome or practitioner? or physician? or program? or programme or programmes or service or services)).ti.
38. (practice and (care or provider?) and (intervention$ or improv$ or project)).ti.
39. (practice and service).ti.

40. individual$ practice.ti.

41. ((practice or practices) and (care or provider? or physician? or doctor? or nurse or nurses) and (intervention$ or improv$ or implement$ or project? or program? or programme or programmes)).ti.

42. ((practice or practices) and (collaborat$ or interdisciplin$ or teambased or team? or mutlidisciplin$ or multi-disciplin$ or crossdisciplin$ or cross-disciplin$)).ti.

43. (25 and (or/26-30)) or (or/31,33-40) [CQI set 3]

44. (quality of health care or quality of care or quality of healthcare).ti,ab.

45. (intervention or implementation).ti.

46. 44 and 45 [CQI Set 4]

47. (improv$ and care).ti.

48. og.fs. [org & administration subheading]

49. 47 and 48 [CQI Set 5]

50. (randomized controlled trial or controlled clinical trial).pt. or randomized.ab. or placebo.ab. or clinical trials as topic.sh. or randomly.ab. or trial.ti.

51. exp animals/ not humans.sh.

52. 50 not 51 [Cochrane RCT Filter 6.4.d Sens/Precision Maximizing]

53. intervention?.ti. or (intervention? adj6 (clinician? or collaborat$ or community or complex or DESIGN$ or doctor? or educational or family doctor? or family physician? or family practitioner? or financial or GP or general practice? or hospital? or impact? or improv$ or individuali?e? or individuali?ing or interdisciplin$ or multicomponent or multi-component or multidisciplin$ or multi-disciplin$ or multifacet$ or multi-facet$ or multimodal$ or multi-modal$ or personali?e? or personali?ing or pharmacies or pharmacist? or pharmacy or physician? or practitioner? or prescrib$ or prescription? or primary care or professional$ or provider? or regulatory or regulatory or tailor$ or target$ or team$ or usual care)).ab.

54. (pre-intervention? or preintervention? or "pre intervention?" or post-intervention? or postintervention? or "post intervention?").ti,ab. [added 2.4]

55. (hospital$ or patient?).hw. and (study or studies or care or health$ or practitioner? or provider? or physician? or nurse? or nursing or doctor?).ti,hw.

56. demonstration project?.ti,ab.

57. (pre-post or "pre test$" or pretest$ or posttest$ or "post test$" or (pre adj5 post)).ti,ab.

58. (pre-workshop or post-workshop or (before adj3 workshop) or (after adj3 workshop)).ti,ab.

59. trial.ti. or ((study adj3 aim?) or "our study").ab.

60. (before adj10 (after or during)).ti,ab.

61. ("quasi-experiment$" or quasiexperiment$ or "quasi random$" or quasirandom$ or "quasi control$" or quasicontrol$ or ((quasi$ or experimental) adj3 (method$ or study or trial or design$))).ti,ab,hw.

62. ("time series" adj2 interrupt$).ti,ab,hw.

63. (time points adj3 (over or multiple or three or four or five or six or seven or eight or nine or ten or eleven or twelve or month$ or hour? or day? or "more than")).ab.

64. pilot.ti.

65. Pilot projects/

66. (clinical trial or controlled clinical trial or multicenter study).pt.

67. (multicentre or multicenter or multi-centre or multi-center).ti.

68. random$.ti,ab. or controlled.ti.

69. (control adj3 (area or cohort? or compare? or condition or design or group? or intervention? or participant? or study)).ab. not (controlled clinical trial or randomized controlled trial).pt.

70. evaluation studies as topic/ or prospective studies/ or retrospective studies/ [Added Jan 2013]

71. (utili?ation or programme or programmes).ti. [Added Jan 2013]

72. (during adj5 period).ti,ab. [Added Jan 2013]

73. ((strategy or strategies) adj2 (improv$ or education$)).ti,ab. [Added Jan 2013]

74. "comment on".cm. or review.pt. or (review not "peer review$").ti. or randomized controlled trial.pt. [Changed Jan 2013]

75. (rat or rats or cow or cows or chicken? or horse or horses or mice or mouse or bovine or animal?).ti.

76. exp animals/ not humans.sh.

77. (or/53-73) not (or/74-76) [EPOC Methods Filter 2.5-added Evaluation Studies line forward--Jan 2013 Medline]

78. ("research support american recovery and reinvestment act" or research support nih extramural or research support nih intramural or research support non us govt or research support us govt non phs or research support us govt phs).pt.

79. (service or services or community or care or intervention or implement$).ti. and (quality.ti,hw. not (*"quality of life"/ or "quality of life".ti.))

80. (bundle? or champion$ or (continuing adj2 education$) or educational or feedback or organi?ation$ or program? or programme or programmes or structured or target$ or training).ti.

81. or/79-80 [Intervention related keywords to focus results]

82. "quality of life".ti. or *Quality of Life/

83. ((or/24,43) not 82) and 52 [RCT: CQI Sets 2 or 3 AND RCT Filter]

84. (and/24,77,81) not (or/82-83) [EPOC-1: CQI Set 2 & EPOC Filter & Intervention Terms]

85. (and/46,78) not (or/82-84) [EPOC-2: CQI Set 4 & Research Support PubType]

86. (49 and (or/43,77-78)) not (or/82-85) [EPOC-3: CQI Set 5 improve care og-fs & (CQI Set 3 or EPOC Filter or Research PT)]

87. (and/43,77,81) not (or/82-86) [EPOC-4: CQI Set 3 & EPOC Filter & Intervention Terms]

88. 3 not (or/75-76) not (or/82-87) [KW: CQI Set 1--keyword results]

Strategy B: May 27, 2013

Ovid MEDLINE(R) In-Process & Other Non-Indexed Citations and Ovid MEDLINE(R) <1946 to Present>

(lean adj (approach or care or enterpri?e or management or method? or methodology or model? or oncology or philosophy or practice or practices or principles or program$ or thinking)).ab. or (lean and (approach or care or enterpri?e or management or method? or methodology or model? or oncology or philosophy or practice or practices or principles or program$ or thinking)).ti. (338)

Strategy C: July 2013

Ovid MEDLINE(R) In-Process & Other Non-Indexed Citations and Ovid MEDLINE(R) <1946 to Present>

1 a list of PMIDs for studies already selected and screened by author team. Run here so that they could be excluded prior to exporting results (194 citations)

2 (lean and (approach or care or enterpri?e or management or method? or methodology or model? or oncology or philosophy or practice or practices or principles or program$ or thinking)).ti. (285)
3 (lean and (process improvement? or performance improvement? or (quality adj2 (care or improv$ or manag$ or outcome? or continuous)) or practice based)).ti. (20)

4 (lean adj2 (approach or care or enterpri?e or management or method? or methodology or model? or oncology or philosophy or practice or practices or principles or program$ or thinking)).ab. (400)

5 (lean adj2 (process improvement? or performance improvement? or (quality adj2 (care or improv$ or manag$ or outcome? or continuous)) or practice based)).ab. (27)

6 (rapid cycle adj3 (approach or approaches or change or collaborat$ or cross-disciplin$ or decision? or healthcare or "health care" or hospital? or impact or initiative? or innovat$ or interprofessional$ or interdisiciplin$ or inter-disciplin$ or management or method$ or optimal$ or organi?ation$ or program? or programme or programmes or process improvement? or project? or quality improvement? or team? or team-based)).ti,ab. (86)

7 or/2-6 [Set 1] (689)

8 exp animals/ not humans.sh. (4002693)

9 (rat or rats or cow or cows or chicken? or horse or horses or mice or mouse or bovine or animal?).ti. (1352098)

10 7 not (or/1,8-9 [Set 1] (380)

11 remove duplicates from 25 (352)

Embase <1974 to 2013 Week 29> OVID

1 (lean and (approach or care or enterpri?e or management or method? or methodology or model? or oncology or philosophy or practice or practices or principles or program$ or thinking)).ti. (384)

2 exp animals/ not humans.sh. (19019080)

3 (rat or rats or cow or cows or chicken? or horse or horses or mice or mouse or bovine or animal?).ti. (1523784)

4 (mass or tissue or adipose or cellular or genet$ or genom$).ti,ab,hw. (5554148)

5 1 not or/2-4 (109) EMBASE

Ovid Healthstar <1999 to June 2013>

Search Strategy:

1 (lean and (approach or care or enterpri?e or management or method? or methodology or model? or oncology or philosophy or practice or practices or principles or program$ or thinking)).ti. (165)

2 exp animals/ not humans.sh. (14460)

3 (rat or rats or cow or cows or chicken? Or horse or horses or mice or mouse or bovine or animal?).ti. (28592)

4 (mass or tissue or adipose or cellular or genet$ or genom$).ti,ab,hw. (808756)

5 1 not or/2-4

EBM Reviews - Health Technology Assessment and EBM Reviews - NHS Economic Evaluation Database = 0

(lean adj3 (approach or care or enterpri?e or management or method? or methodology or model? or oncology or philosophy or practice or practices or principles or program$ or thinking)).mp. or (lean and (approach or care or enterpri?e or management or method? or methodology or model? or oncology or philosophy or practice or practices or principles or program$ or thinking)).ti.

Web of Science

Databases=SCI-EXPANDED, SSCI, A&HCI, CPCI-S, CPCI-SSH Timespan=All years

Search 1: Title=("rapid cycle" or "plan do study" or "plan do check" or (shewhart near/3 cycle) or (shewhart* near/3 method*) or (deming near/2 management) or (deming near/2 philosoph*) or (deming near/2 principle*) or (deming near/3 method*) or "breakthrough series" or "breakthrough collaborative*" or "breakthrough project") AND Topic=("health care" or "primary care" or healthcare or hospital or hospitals) AND Document Types=(ARTICLE OR MEETING ABSTRACT OR PROCEEDINGS PAPER)= 16

Search 2: Title=("rapid cycle" or "plan do study" or "plan do check" or (shewhart near/3 cycle) or (shewhart* near/3 method*) or (deming near/2 management) or (deming near/2 philosoph*) or (deming near/2 principle*) or (deming near/3 method*) or "breakthrough series" or "breakthrough collaborative*" or "breakthrough project") AND **Topic=("**health care" or "primary care" or healthcare or hospital or hospitals) AND *Databases=SCI-EXPANDED, SSCI, A&HCI, CPCI-S, CPCI-SSH Timespan=All years = 19*

Search 3: Title=("rapid cycle" or "plan do study" or "plan do check" or (shewhart near/3 cycle) or (shewhart* near/3 method*) or (deming near/2 management) or (deming near/2 philosoph*) or (deming near/2 principle*) or (deming near/3 method*) or "breakthrough series" or "breakthrough collaborative*" or "breakthrough project") AND **Title=("**health care" or "primary care" or healthcare or hospital or hospitals) = 4
Databases=SCI-EXPANDED, SSCI, A&HCI, CPCI-S, CPCI-SSH Timespan=All years

Search 4: TI=(lean) AND TI=(management OR system OR systems OR approach OR method* OR principle* OR model* OR thinking) AND TS=("health care" or "primary care" or healthcare or hospital or hospitals) = 59

Search 5: Title=(lean) AND Title=(management OR system OR systems OR approach OR method* OR principle* OR model* OR thinking) AND Title=("health care" or "primary care" or healthcare or hospital or hospitals) AND Document Types=(ARTICLE OR MEETING ABSTRACT OR PROCEEDINGS PAPER) = 16

ProQUest Platform; multiple databases searched using the following strategy:

("lean system*" or "lean approach" or "lean management" or "lean method*" or "lean practice" or "lean principle*" or "lean model*" or "lean practices" or "lean thinking" or "concept map*" or "process map*" or "value stream map*" or "fault tree analysis" or "failure mode and effects analysis" or "rapid cycle" or "plan do study" or "plan do check" or (shewhart near/3 cycle) or (shewhart* near/3 method*) or (deming near/2 management) or (deming near/2 philosoph*) or (deming near/2 principle*) or (deming near/3 method*) or "breakthrough series" or "breakthrough collaborative*" or "breakthrough project") AND ("health care" or healthcare or medicine or medical or disease management or "hospital" or "hospitals" or "primary care" or managed care or medicaid or medicare)

Dissertations & Theses (searched in ti/ab) = 120

PAIS International = 30

ProQuest Political science collection = 6

Canadian Research Index = 1 potentially relevant of 10 (M. Fiander screened)

Grey Literature

Rewarding Provider Performance : Aligning Incentives in Medicare (Pathways to Quality health Care Series, 2007. The Nation Academy of Science Press. Report available online: http://www.nap.edu/catalog.php?record_id=11723

Suggested authors browse:

AHRQ Innovations Exchange. Articles & Guides: http://innovations.ahrq.gov/articles.aspx May be worth browsing if you have not done so. http://innovations.ahrq.gov/innovations_qualitytools.aspx ASQ Web site: http://asq.org/healthcaresixsigma/articles/ssfhc-form.html
