# Supplementary material for: Lean management in health care: definition, concepts, methodology and effects reported (systematic review protocol)
Source: Syst Rev. 2014 Sep 19;3:103. doi: 10.1186/2046-4053-3-103 (PMC4171573; doi:10.1186/2046-4053-3-103)
Supplement: Additional file 2 — EPOC risk of bias criteria. This file contains the suggested risk of bias for EPOC reviews. [file 2046-4053-3-103-S2.pdf]

Suggested risk of bias criteria for EPOC reviews

Risk of bias for studies with a separate control group

Randomised controlled trials (RCTs)
Non-randomised contolled trials (NRCTs)
Controlled before-after (CBA) studies

Nine standard criteria are suggested for all RCTs, NRCTs and CBA studies. Further information can be obtained from the Cochrane handbook section on risk of bias.

Was the allocation sequence adequately generated?
Score "Low risk" if a random component in the sequence generation process is described (eg Referring to a random number table). Score "High risk" when a nonrandom method is used (eg performed by date of admission). NRCTs and CBA studies should be scored "High risk". Score "Unclear risk" if not specified in the paper.

Was the allocation adequately concealed?
Score "Low risk" if the unit of allocation was by institution, team or professional and allocation was performed on all units at the start of the study; or if the unit of allocation was by patient or episode of care and there was some form of centralised randomisation scheme, an on-site computer system or sealed opaque envelopes were used. CBA studies should be scored "High risk". Score "Unclear risk" if not specified in the paper.

Were baseline outcome measurements similar?[1,2]
Score "Low risk" if performance or patient outcomes were measured prior to the intervention, and no important differences were present across study groups. In RCTs, score "Low risk" if imbalanced but appropriate adjusted analysis was performed (e.g. Analysis of covariance). Score "High risk" if important differences were present and not adjusted for in analysis. If RCTs have no baseline measure of outcome, score "Unclear risk".

Were baseline characteristics similar?
Score "Low risk" if baseline characteristics of the study and control providers are reported and similar. Score "Unclear risk" if it is not clear in the paper (e.g. characteristics are mentioned in text but no data were presented). Score "High risk" if there is no report of characteristics in text or tables or if there are differences between control and intervention providers. Note that in some cases imbalance in patient characteristics may be due to recruitment bias whereby the provider was responsible for recruiting patients into the trial.

---

[1] If some primary outcomes were imbalanced at baseline, assessed blindly or affected by missing data and others were not, each primary outcome can be scored separately.

[2] If "Unclear risk" or "High risk", but there is sufficient data in the paper to do an adjusted analysis (e.g. Baseline adjustment analysis or Intention to treat analysis) the criteria should be re scored as "Low risk".

Were incomplete outcome data adequately addressed?[1]

Score "Low risk" if missing outcome measures were unlikely to bias the results (e.g. the proportion of missing data was similar in the intervention and control groups or the proportion of missing data was less than the effect size i.e. unlikely to overturn the study result). Score "High risk" if missing outcome data was likely to bias the results. Score "Unclear risk" if not specified in the paper (Do not assume 100% follow up unless stated explicitly).

Was knowledge of the allocated interventions adequately prevented during the study?[1]

Score "Low risk" if the authors state explicitly that the primary outcome variables were assessed blindly, or the outcomes are objective, e.g. length of hospital stay. Primary outcomes are those variables that correspond to the primary hypothesis or question as defined by the authors. Score "High risk" if the outcomes were not assessed blindly. Score "Unclear risk" if not specified in the paper.

Was the study adequately protected against contamination?
Score "Low risk" if allocation was by community, institution or practice and it is unlikely that the control group received the intervention. Score "High risk" if it is likely that the control group received the intervention (e.g. if patients rather than professionals were randomised). Score "Unclear risk" if professionals were allocated within a clinic or practice and it is possible that communication between intervention and control professionals could have occurred (e.g. physicians within practices were allocated to intervention or control)

Was the study free from selective outcome reporting?
Score "Low risk" if there is no evidence that outcomes were selectively reported (e.g. all relevant outcomes in the methods section are reported in the results section). Score "High risk" if some important outcomes are subsequently omitted from the results. Score "Unclear risk" if not specified in the paper.

Was the study free from other risks of bias?
Score "Low risk" if there is no evidence of other risk of biases

Risk of bias for interrupted time series (ITS) studies

Seven standard criteria are used for all ITS studies. Further information can be obtained from the Cochrane handbook section on Risk of Bias and from the draft methods paper on risk of bias under the EPOC specific resources section of the EPOC website.

Note: If the ITS study has ignored secular (trend) changes and performed a simple t-test of the pre versus post intervention periods without further justification, the study should not be included in the review unless reanalysis is possible.

Was the intervention independent of other changes?
Score "Low risk" if there are compelling arguments that the intervention occurred independently of other changes over time and the outcome was not influenced by other confounding variables/historic events during study period. *If Events/variables identified, note what they are.* Score "High risk" if reported that intervention was not independent of other changes in time.

Was the shape of the intervention effect pre-specified?
Score "Low risk" if point of analysis is the point of intervention OR a rational explanation for the shape of intervention effect was given by the author(s). Where appropriate, this should include an explanation if the point of analysis is NOT the point of intervention; Score "High risk" if it is clear that the condition above is not met.

Was the intervention unlikely to affect data collection?

Score "Low risk" if reported that intervention itself was unlikely to affect data collection (for example, sources and methods of data collection were the same before and after the intervention); Score "High risk" if the intervention itself was likely to affect data collection (for example, any change in source or method of data collection reported).

Was knowledge of the allocated interventions adequately prevented during the study?[3]

Score "Low risk" if the authors state explicitly that the primary outcome variables were assessed blindly, or the outcomes are objective, e.g. length of hospital stay. Primary outcomes are those variables that correspond to the primary hypothesis or question as defined by the authors. Score "High risk" if the outcomes were not assessed blindly. Score "Unclear risk" if not specified in the paper.

---

[3] If some primary outcomes were assessed blindly or affected by missing data and others were not, each primary outcome can be scored separately.

Were incomplete outcome data adequately addressed?[3]

Score "Low risk" if missing outcome measures were unlikely to bias the results (e.g. the proportion of missing data was similar in the pre- and post-intervention periods or the proportion of missing data was less than the effect size i.e. unlikely to overturn the study result). Score "High risk" if missing outcome data was likely to bias the results. Score "Unclear risk" if not specified in the paper (Do not assume 100% follow up unless stated explicitly).

Was the study free from selective outcome reporting?
Score "Low risk" if there is no evidence that outcomes were selectively reported (e.g. all relevant outcomes in the methods section are reported in the results section). Score "High risk" if some important outcomes are subsequently omitted from the results. Score "Unclear risk" if not specified in the paper.

Was the study free from other risks of bias?
Score "Low risk" if there is no evidence of other risk of biases. e.g. should consider if seasonality is an issue (i.e. if January to June comprises the pre-intervention period and July to December the post, could the "seasons' have caused a spurious effect).
